# Supplementary material for: Central nervous system hemangioblastomas in von Hippel-Lindau disease: Total growth rate and risk of developing new lesions not associated with circulating VEGF levels
Source: PLoS One. 2022 Nov 28;17(11):e0278166. doi: 10.1371/journal.pone.0278166 (PMC9704563; doi:10.1371/journal.pone.0278166)
Supplement: S1 File — (DOCX) [file pone.0278166.s001.docx]

*Antibodies and oligonucleotides*

Affinity-purified polyclonal biotinylated antibodies against VEGF_165_ were procured from R&D Systems (BAF293). Streptavidin-conjugated Biovic3 and Biovic5 were purchased from Avidomics. The forward primer (Biofwd), reverse primer (Biorev) and connector oligonucleotide were obtained from Integrated DNA Technologies (IDT). Details for all oligonucleotides used in this paper are summarized in Supplementary Table 1.

*Immobilization of antibodies on microparticle beads*

Biotinylated antibodies were immobilized on the Dynabeads MyOne T1 streptavidin coated beads (Thermo Fisher). In order to immobilize biotinylated antibodies on the beads, 1 mg of Dynabeads MyOne T1 was first washed twice with 500 μl, of washing buffer (PBS supplemented with 0.05% Tween-20). Then, the beads were mixed with 200 μl of 50 nM biotinylated antibodies and incubated for 1 hour at RT. Thereafter, the beads were washed twice, and reconstituted with 200 μl of storage buffer (PBS supplemented with 0.1% Bovine Serum Albumin (BSA)).

*PLA probes preparation*

The probes were functionalized by conjugating single-stranded DNA molecules of approximately 60 nucleotides in length to antibodies via biotin-streptavidin conjugation.

*Biotin-streptavidin conjugation*

For the preparation of PLA probes using streptavidin modified oligonucleotides, biotinylated antibodies, were separately mixed with 100 nM streptavidin-oligonucleotides (streptavidin-Biovic3 and streptavidin-Biovic5) at a 1:1 molar ratio. These mixtures were incubated for 1 hour at RT. The antibody-oligonucleotide pairs were then combined to a 500 pM final concentration in PLA buffer (1mM D-biotin (Invitrogen), 0.1% BSA (New England Biolabs), 0.05% Tween-20 (Sigma Aldrich), 100 nM goat IgG (Sigma-Aldrich), 0.1 μg/μl salmon sperm DNA (Invitrogen), 5 mM EDTA, PBS), before being used in the assay.

*Solid-phase PLA for detection of PTMs*

Solid-phase PLA was performed as described previously(13). For each PLA reaction, the storage buffer of 1μl of antibody-coated microparticle beads was replaced by 5μl of PLA buffer (1mM D-biotin (Invitrogen), 0.1% BSA (New England Biolabs), 0.05% Tween-20 (Sigma Aldrich), 100nM goat IgG (Sigma-Aldrich), 0.1μg/μl salmon sperm DNA (Invitrogen), 5mM EDTA, 1x PBS) before being mixed with the sample. A dilution series of the antigens were prepared in PLA buffer and 10% chicken serum (Invitrogen), and all included negative controls where no protein was added to determine the background noise of the assay. The assays commenced with the mixing of 45μl of each sample with the 5μl of the microparticle beads in microtiter wells and incubated for 1-1.5 h at RT under rotation. After the incubation, the beads were then washed twice with washing buffer, and 50μl of PLA probe mixture at concentration of 500pM for each probe was added to each well, and incubated for an additional 1-1.5 hours at RT under rotation. Subsequently, the microparticle beads were washed twice with washing buffer and 50μl of qPCR master mix (1x PCR buffer, 2.5mM MgCl2 (Invitrogen), 0.1μM concentration of each primer (Biofwd and Biorev) and connector oligonucleotide (Biosplint), 0.5X Sybr Green (Thermo Fischer Scientific), 0.08mM ATP, 0.2mM dNTPs (containing dUTP), 1.5 units of Platinum *Taq* polymerase, 0.02 units of T4 DNA ligase (30U/μl) (Sigma-Aldrich), 0.1 unit of uracil –DNA glycosylase (1U/μl)(Thermo Fischer Scientific)) were added to each well, and followed by detection of ligation product via qPCR performed on ABI 7900 (Thermo Fischer Scientific). The thermocycling program used for the detection of signal was a 40-cycle program, which includes 2 min incubation at 95^o^C, followed by 40 cycles of 15 seconds at 95^o^C and 1 minute at 60 ^o^C.

*Data analysis*

The recorded cycle treshold values for q-PCR data were further analyzed with Microsoft Excel. In addition, ImageJ software was used in the results analysis to determine limit of detection (LOD), lowest limit of quantification (LLOQ) and dynamic ranges for all assays. The LOD for the SP-PLAs was defined as the concentration of protein corresponding to Ct_LOD_ = Ct_N_ – (2 x S_N_), where Ct_N_ is the average Ct acquired for the background noise, and S_N_ is the standard deviation of that value. The LLOQ for SP-PLA was defined as Ct_N_ – (10 x S_N_); while for ELISA it was OD_N_ + (10 x S_N_).
