# Supplementary material for: Central nervous system hemangioblastomas in von Hippel-Lindau disease: Total growth rate and risk of developing new lesions not associated with circulating VEGF levels
Source: PLoS One. 2022 Nov 28;17(11):e0278166. doi: 10.1371/journal.pone.0278166 (PMC9704563; doi:10.1371/journal.pone.0278166)
Supplement: Attachment — Submitted filename: Rebuttal letter for revision of PONE.docx [file pone.0278166.s002.docx]

*Rebuttal letter for revision of PONE-D-22-00869*

*Central nervous system hemangioblastomas in von Hippel-Lindau disease: total growth rate and risk of developing new lesions not associated with circulating VEGF levels*

We wish to thank the reviewers for their thorough and insightful review of our paper. Please see our answers to the points raised below.

Regarding reviewer #1

As commented in the manuscript, we also would like to assess VEGF levels in CSF of vHL patients in the future. The nature of the lesions may make this somewhat difficult, but we hope to be able to address this in the future.

The tumor volume change in fig 1 regarding patient 14 (we presume that the 4 mentioned applies to pt 14, since pat 4 is not included in this analysis due to lack of follow-up investigations) is due to surgical intervention. This is hopefully made clearer in the figure legend in the revised manuscript. Since the total tumor volume is an absolute value, no SD is applicable. Gender has been added to the pt numbers in the figure.

Regarding reviewer #2

1. The PLA method was set up by our lab to possibly increase throughput. It was previously validated using an ELISA kit, and select samples in this study were analyzed with an ELISA with similar results.
2. This study was designed to address whether the total CNS tumor volume is reflected in circulating VEGF. We will hopefully be able to continue working and address this very relevant question in the future, but a clinically useful biomarker should reflect the tumor burden at a single time point.
3. Volumetric segmentation was used. This has been made clearer in the methods section.
4. This mistake has been corrected.
5. The statement has been amended. References to relevant articles (already cited) has been added.
6. The VEGF sampling was done at initial visit (year 1). This has been added to the figure legend.
7. The figure has been adjusted accordingly.

Regarding reviewer #3

The critique regarding the small number of patients is relevant, and we wish to start collaborating with other centers to increase the sample size in the future.

In the revised manuscript, the details regarding the laboratory methods have been somewhat shortened. We have also rewritten and expanded on the radiology portion of the methods section. We have also performed volumetry of associated tumor cysts in patients harboring these lesions and investigated whether total tumor volume including cystic parts correlates with VEGF levels. A new figure has been added and the results are presented in the text. Since no correlation was seen this does not change the message of the manuscript. Although clinically important, volumetric measurement of edema is not technically reliable enough to include.

The diagnostic criteria of multiple hemangioblastomas for vHL was mentioned in the introduction (more than one vHL-associated lesion), but we have highlighted this instance further.

The genetic data has been updated as suggested.

The results section has been revised and will now hopefully read in a more fluent way.

We hope that these comments and the revisions made have been satisfactory. We look forward to answering any further comments or questions you may have regarding the manuscript if further revision is necessary.

On behalf of all co-authors,

Dr Jimmy Sundblom
