# Supplementary material for: Central nervous system hemangioblastomas in von Hippel-Lindau disease: Total growth rate and risk of developing new lesions not associated with circulating VEGF levels
Source: PLoS One. 2022 Nov 28;17(11):e0278166. doi: 10.1371/journal.pone.0278166 (PMC9704563; doi:10.1371/journal.pone.0278166)
Supplement: Attachment — Submitted filename: Rebuttal letter for revision of PONE New.docx [file pone.0278166.s003.docx]

*Rebuttal letter for revision of PONE-D-22-00869*

*Central nervous system hemangioblastomas in von Hippel-Lindau disease: total growth rate and risk of developing new lesions not associated with circulating VEGF levels*

We wish to thank the reviewers again for their thorough and insightful review of our paper. Please see our answers to the points raised below.

Reviewer #3: The manuscript writing improved significantly, although a few minor issues remained.
My main criticism for the study is yet in its core hypothesis – most of the VEGF in the circulation derive from peripheral blood cells, and the chances to see hemangioblastoma-derived VEGF in a meaningful concentration is extremely low. To prove that, even comparing patients with various VHL-related, VEGF-dependent tumors, to controls, shown no difference.
In my view, the authors might want to discuss this point in the discussion.

-The discussion has been updated and references added to stress this important issue.

In addition, please revise the variants descriptions in Table 1 – the 167 codons is described as nc499 in VHL011 and nc712 in VHL012.

-This oversight has been corrected

Minor
Editing, typos – therefor -> therefore, missing full stop in the abstract, changing fonts in the abstract.
Genetic analysis – VHL gene should be written in capital letters and in Italics.

-These typos and others has been amended.

We hope that these comments and the revisions made have been satisfactory. We look forward to answering any further comments or questions you may have regarding the manuscript if further revision is necessary.

On behalf of all co-authors,

Dr Jimmy Sundblom
